# A Symposium: Otitis Media Suppurativa

**Published:** 1903-07

**Authors:** 

**Affiliations:** Brownwood, Texas


### Eye, Ear, Nose and Throat.

P. M. PAYNE, M. D., EDITOR, BROWNWOOD, TEXAS.

### "A Symposium: Otitis Media Suppurativa."

The above caption is the title of a series of papers appearing in the June *Laryngoscope*.

The papers were all read at the ninth annual meeting of the American Laryngological, Rhinological and Otological Society, at Lexington, Kentucky, May 1, 1903.

The contributors are all men of national and international reputation: Dr. Norval H. Pierce, of Chicago; Dr. S. MacCuen Smith, of Philadelphia; Dr. Chas. W. Richardson, of Washington, D. C.; Dr. James F. McKernon, of New York; and Dr. Edward B. Dench, of New York.

Dr. Pierce says, in the part under "Age": "The greatest number of suppurative ear diseases occur in the first three years of life. The predisposition of children to otic infection is due to hyperplasia of the lymphoid structures in the pharynx, and post-nasal space, and to the frequency with which the acute exanthemata occur at this point."

His paper deals only with the etiology, pathology and symptomatology of the disease.

Dr. S. MacCuen Smith lays stress on the great importance of an early recognition of the disease by the general practitioner, and says: "The ignorance of the laity in the most fundamental rules governing the care of their own ears, and especially those of their children, is not surprising when we recall the empiricism that has surrounded the organ of hearing and its treatment throughout the history of medicine." He further says, regarding the training of students: "I, for one, regard it as a disgrace and an injustice, both to the embryo physician and to the public, to confer the honored degree of doctor of medicine upon any candidate who is not able to diagnose and properly treat acute diseases of the tympanic cavity."

Under "Treatment," he has the following to say: "In considering the treatment of acute suppurative otitis media it must be assumed that every effort has been made to arrest the progress of the disease before the formation of pus. If, however, pus formation has already taken place, there is, of course, nothing to do but evacuate it in the manner above stated," which is, "when the middle ear, as well as other cavities, is filled with pus, it must be thoroughly evacuated; this, however, can not be accomplished by a simple puncture of the membrana tympani. The membrane, consequently, must be *incised freely*, the chief requisite being to carry the incision from the most bulging point downward to the lower border of the canal, said incision to be continued either in an anterior or posterior direction until about the sixteenth part of a circle has been formed. This will not only provide for good drainage, but will insure the opening remaining patulous long enough to admit of after treatment."

Dr. Richardson's paper was on "The Etiology, Pathology and Symptomatology of Chronic Suppurative Otitis."

Dr. McKernon's paper dealt with the "Treatment of Complications of Otitis Media Suppurativa," and is one of the most interesting papers in the collection. One, however, should read it all to appreciate it thoroughly.

Dr. Edward Bradford Dench described the "Technique of the Radical Operation for Chronic Suppurative Otitis Media." To attempt to extract from his paper would be an injustice to the author, as each step fits in to the one preceding.

These five articles alone are worth a year's subscription to the *Laryngoscope.*

---

The writer recently spent a few weeks in New Orleans, and while there visited daily the clinics at the Eye, Ear, Nose and Throat Hospital. They are treating on an average, daily, about 150 to 200 patients, and will soon erect a handsome new hospital with every modern equipment.

A grandson of our immortal Stephen F. Austin was in the hospital and had been operated on by Dr. Gordan King, chief of clinic of the Ear, Nose and Throat Department, for a large benign tumor of the naso-pharynx. Recovery was complete, and the young boy returned to his home in Lindale, Smith county, Texas, while the writer was still there.

